# Twelfth Annual ENBDC Workshop: Methods in Mammary Gland Biology and Breast Cancer

**DOI:** 10.1007/s10911-021-09498-z

**Published:** 2021-08-26

**Authors:** Elsa Charifou, Gunnhildur Asta Traustadottir, Mohamed Bentires-Alj, Beatrice Howard, Alexandra Van Keymeulen

**Affiliations:** 1 Cellular Plasticity and Disease Modeling, Department of Developmental & Stem Cell Biology, CNRS UMR3738 - Institut Pasteur, 25 rue du Dr Roux, 75015 Paris, France; 2 Stem Cell Research Unit, Department of Anatomy, Faculty of Medicine, School of Health Sciences, Biomedical Center, University of Iceland, Reykjavík, Iceland; 3 Department of Biomedicine, University of Basel, University Hospital Basel, Basel, Switzerland; 4 The Breast Cancer Now Toby Robins Research Centre, The Institute of Cancer Research, London, UK; 5 Laboratory of Stem Cells and Cancer, Université Libre de Bruxelles (ULB), Brussels, Belgium

**Keywords:** Mammary gland, Breast cancer, Breast development, Branching, Organoids, Patient-derived xenografts, Circulating tumor cells, Metastasis, Tumor dormancy, Tumor microenvironment, Signaling, Premetastatic niche, In vivo live imaging, Lineage tracing, Transcriptomics, Resistance to therapy

## Abstract

The twelfth annual workshop of the European Network for Breast Development and Cancer focused on methods in mammary gland biology and breast cancer, was scheduled to take place on March 26–28, 2020, in Weggis, Switzerland. Due to the COVID-19 pandemic, the meeting was rescheduled twice and eventually happened as a virtual meeting on April 22 and 23, 2021. The main topics of the meeting were branching and development of the mammary gland, tumor microenvironment, circulating tumor cells, tumor dormancy and breast cancer metastasis. Novel and unpublished findings related to these topics were presented, with a particular focus on the methods used to obtain them. Virtual poster sessions were a success, with many constructive and fruitful interactions between researchers and covered many areas of mammary gland biology and breast cancer.

## Introduction

The European network of breast development and cancer (ENBDC) organizes an annual workshop on methods in mammary gland biology and breast cancer. This three-day meeting has been hosted every year until now in the charming small town Weggis in Switzerland [1], but the twelfth annual meeting took place virtually on April 22 and 23, 2021. The virtual format allowed many more scientists to attend the meeting and we reached over 100 participants from many different countries, while the ENBDC meeting on site usually gathers around 65 participants [1]. The meeting schedule was rearranged to fit in two full days, with regular breaks, and more relaxed poster sessions in the afternoon. This first virtual meeting was a great success, with high quality talks on very hot topics and discussions after each talk. Poster sessions enabled all attendees to present their work to a smaller audience. The conviviality and friendly interactions we usually enjoy during breaks and meals in Weggis were however missed by most of us, and we are all looking forward to gather next year in Weggis on April 28–30, 2022.

## Meeting Report

The meeting started with the Keynote speaker Nicola Aceto, from the Swiss Federal Institute of Technology Zürich (ETH) in Switzerland. Nicola Aceto’s work focuses on circulating tumor cells (CTC), some of which will eventually lead to metastasis. He started his presentation by explaining the size-based, antigen-independent microfluidic technique used to trap CTC which are bigger and less deformable than blood cells [2]. Nicola Aceto then described the presence of CTC clusters [3] and their characteristics and showed how these CTC clusters in patients are associated with a worse prognosis. Using mice engrafted with tagged mammary tumor cells, he demonstrated that these CTC clusters had a 50-fold increased metastatic potential compared to single CTC [4–6]. He went further in the comparison of single and clustered CTC and showed that the latter bear hypomethylated DNA regions and featured stem and proliferative-like programs, expressing *OCT4*, *SOX2* and *NANOG*. He then presented an elegant idea to screen for drugs able to dissociate clustered CTC without killing the cells, and found that a Na + K + ATPase inhibitor presented these characteristics. Validation in vivo in mice showed that this inhibitor dissociated CTC clusters without killing single CTC and decreased the metastatic index [7]. Ongoing effort is focused on translating this work to human patients in ongoing clinical trials. Another characteristic of clustered CTC is that some of them include immune cells. He showed that breast cancer patient survival was worst when they present CTC white blood cell (WBC) clusters. Using single cell RNA sequencing of patient samples, he found that in 90% of the cases these WBC were neutrophils. In mice, he showed that CTC-neutrophil clusters were the most metastasis-competent CTC subpopulation [8]. He concluded this talk presenting his work on hypoxia, which he showed is not restricted to the center of the tumor. Intratumor hypoxia leads to cell–cell junction upregulation. Clustered CTC were usually hypoxic, while single CTC were normoxic. Anti-angiogenic therapy reduced tumor size, but increases hypoxia, CTC clusters and metastasis [9]. This observation will be important to keep in mind when testing anti-cancer therapies.

The first session focused on breast cancer metastasis and was chaired by Mohamed Bentires-Alj from University of Basel, Switzerland. The first speaker of the session was Ilaria Malanchi form the Francis Crick Institute in London, UK. Her work focuses on the local interaction of metastatic cells within the tissue. To study this technically challenging question, she set up a new method called Cherry-niche which enables cells expressing a fluorescent protein to label surrounding cells [10, 11]. In particular, she focused her study on the metastatic niche in the mouse lung, using injection of 4T1 mouse mammary metastatic cells. Using the Cherry-niche system, cancer cells express *GFP* and a liposoluble red fluorescent tag and appear yellow, while neighboring cells, which constitute the niche, are tagged with the red fluorescent compound and the rest of the lung is not labelled. With this elegant approach, she showed the presence of cancer associated parenchymal cells with stem cell-like features and expression of lung progenitor markers, as well as self-renewal and differentiation potential [10].

Eva Gonzalez-Suarez, from the Spanish National Cancer Research Center (CNIO) in Spain, was the second speaker of this session, and presented her work on *RANK* and *RANKL*. She showed that loss of *RANK* signaling in *MMTV-PyMT* mouse tumor cells increased the number of lymphocytes, leukocytes and CD8 + T cells, and reduced macrophage and neutrophil infiltration, clearly demonstrating an effect of *RANK* pathway on mammary tumor immune surveillance. She showed that *RANKL* inhibition increased the effect of immunotherapy in mammary gland tumors. This immune-modulatory effect of *RANK* signaling (increased TILS and CD8 + T cells) was confirmed in pre-menopausal early breast cancer patients treated with denosumab in the D-BEYOND trial and is being further investigated in an ongoing clinical trial (D-BIOMARK) treating pre- and post-menopausal women with early breast cancer with neoadjuvant denosumab. Her findings indicate that tumor cells use *RANK* pathway to evade immune surveillance and support the use of *RANK* pathway inhibitors to increase response to immunotherapy in luminal breast cancer [12]. She presented new results showing that *RANK* expression unexpectedly delayed mammary tumor latency in two oncogene-driven mouse models, (*MMTV-PyMT* and *MMTV-Neu*). The mechanism behind this observation is that activation of *RANK* signaling induced senescence through *P16/P19*. This *RANK*-induced senescence increased stemness properties and promoted tumor growth and metastasis. This work shows that while *RANK* induces senescence and stemness, which delays tumor initiation, it eventually increases tumor aggressiveness [13].

Roger Gomis, from the Institute for Research in Biomedicine (IRB) Barcelona in Spain, presented a short talk on metastasis latency and bone metastasis in breast cancer. His work demonstrated that gain in a chromosomal region coding for the transcription factor *MAF* in primary breast tumors is associated with metastasis in bone but not to other organs [14]. *MAF* is therefore a potential biomarker to select patients at risk of bone metastasis for bisphosphonate adjuvant therapy. To go further on this important observation, a clinical trial was performed and concluded that *MAF* amplification status predicts likelihood of benefit from adjuvant zoledronic acid [14, 15]. Roger Gomis is now focusing on the *MAF*-mediated metastasis mechanisms and is studying *MAF* interactome as well as the *MAF*-mediated chromatin opening and transcriptional program.

Ana Luisa Correia, a postdoc from Mohamed Bentires-Alj lab at the University of Basel in Switzerland, closed this session on breast tumor dormancy and metastasis with a short talk on her recent findings. Her work focuses on dormant disseminated tumor cells (DTC). In order to study the differences between cycling and quiescent DTCs, she used mouse models of metastasis consisting of xenografting human MDA-MB-231 cells into the mammary glands of immunocompromised *NOD-SCID* mice, followed by primary tumor resection. The novelty of her experimental approach is in using tumor cells co-expressing a *Tomato* reporter and a mutant reporter of *P27* which identifies quiescent cells. Analysis of the distribution of DTCs amongst different metastatic organs showed that the liver, often associated with poor prognosis, mainly harbored DTCs in dormant state. She showed that there was a selective increase in natural killer (NK) cells in the dormant microenvironment. She then showed in mice that immunotherapy ensured a pool of NK cells that sustained dormancy through interferon gamma signaling, which appeared to be a master switch between dormancy and metastasis in the liver. Lastly, Ana found that liver injury limits NK cell expansion. Using a model of chemically-induced liver injury, she showed that hepatic stellate cells become activated and secrete an inhibitor of NK cell proliferation, *CXCL12*, through its cognate receptor *CXCR4*. This study is very promising as it suggests that therapies aiming at normalizing NK cell density could halt dormant DTCs from awakening and prevent metastatic disease [16].

We were pleased to listen to John Stingl next, a former member of the ENBDC committee now working at STEMCELL Technologies Inc. in Canada, for the Meet the Expert session. STEMCELL is commercializing media for growing organoids from a wide variety of tissues, including the mouse mammary gland. John shared with us his protocols and advice on how to culture mammary gland organoids in Matrigel domes with the MammoCult Organoid Growth Medium (OGM) developed at STEMCELL. An important trait of the MammoCult OGM is that it is serum-, estrogen-, progesterone- and phenol red-free. Different combinations of supplements are used, depending on whether branched multilineage or luminal-restricted organoids are desired. John recommended that for maintaining organoid lines they were passaged as fragments since this would help maintain a normal karyotype, however seeding cells as single cells was recommended for quantitative experiments since there would be less variability in organoid numbers between technical replicates. Branched organoids are multilineage, with basal cells expressing *K14* and *SMA*, and luminal cells expressing *K8* and *PR*. These organoids were able to synthesize casein in response to lactogenic stimulation. He also presented data on EpiCult Plus Medium, which is a serum-free medium that promotes robust long-term expansion of mouse mammary cells in 2D culture in the absence of feeders, while still retaining organoid-forming potential. This medium could be useful for gene editing prior to organoid culture.

The second day, we moved on with the branching and mammary development session, chaired by Beatrice Howard from The Institute of Cancer Research, UK. Our first speaker was Thorarinn Gudjonsson from the University of Iceland, Iceland, with his talk on epithelial to mesenchymal transition (EMT) in breast morphogenesis and cancer. He presented the generation of the D492 cell line by isolating MUCIN 1 negative, EPCAM positive cells from primary cultures of breast cells obtained from reduction mammoplasties. This cell line has epithelial characteristics as well as stem cell properties and is able to form branched 3D structures resembling terminal duct lobular units [17, 18]. By co-culturing D492 cells and primary breast endothelial cells in Matrigel, he showed that endothelial cells induced branching morphogenesis, highlighting the importance of endothelial cells in branching morphogenesis, but also that some colonies showed spindle shape with EMT characteristics. A new cell line, named D492M, was derived from these spindle colonies, and showed mesenchymal traits [19]. The two cell lines, D492 with epithelial properties and D492M with mesenchymal properties, allowed to study EMT and mesenchymal to epithelial transition (MET). Comparison of microRNA profiling of the two cell lines indicated that *miRNA 200c/miRNA 141* and *miRNA 203* were down-regulated in D492 cells and that overexpression of these miRNA in D492M cell line reinduced epithelial traits, demonstrating their role in EMT and MET. In particular, overexpression of *miRNA 200c/miRNA 141* combined with *p63* was able to completely reverse the mesenchymal phenotype to a fully branched epithelial phenotype [20]. The D492 cell line was also used to generate a tumorigenic cell line expressing *HER2* for use in breast cancer studies [21].

The next speaker was Bethan Lloyd-Lewis, who recently established her lab at the University of Bristol, UK. She presented her previous postdoctoral work where she used intravital imaging to investigate mammary epithelial cell fate dynamics during normal and pathological mammary morphogenesis, alongside developing a flexible and suture-less, silicone-based imaging window for rodent intravital microscopy. This low-cost, suture-less device is suitable for many anatomical locations, representing a substantial advance the field  [22]. Using intravital imaging in *NOTCH1-CREERT2* and *SMA-CREERT2* lines crossed to *mT/mG* fluorescent reporter mice, she traced luminal and basal mammary cell behaviors during ductal development, and in response to mutagenic beta-catenin activation. This approach revealed that, regardless of the mammary lineage targeted, mutant beta-catenin stabilisation leads to cellular rearrangements that precipitate the formation of hyperplastic lesions that undergo squamous transdifferentiation [23].

Satu-Marja Myllymäki, a postdoc from Marja Mikkola’s lab in Helsinki Institute of Life Science (HiLIFE), Institute of Biotechnology in Helsinki, Finland, presented a short talk entitled “From cells to branches- Insights into mammary branch formation”. While there are two mechanisms leading to branching: tip bifurcation and side branching, there are many behaviors that can drive branching like localized cell proliferation, oriented cell division, directional migration, cell rearrangement, cell shape change or adhesion remodeling. She focused her work on the contribution of localized cell proliferation and directional migration. Using the *Fucci2a* mice [24], in which cells in M/G2/S phase are green and cells in G1/GO are red, she showed that luminal cells were more frequently detected in M/G2/S phase than basal cells, and that luminal cells in tips were more frequently detected in M/G2/S phase than luminal cells in duct and branch points. Mikkola lab used an ex vivo culture system of embryonic mammary glands to study branching events and quantified the proportion of lateral branching, bifurcations and trifurcations. She presented her data on how cell proliferation and cell motility contributed to branching. Her data suggest that establishment of a new branch point during bifurcation may require cell immobilization and inhibition of cell cycle activity and that proliferation becomes restricted to the daughter tips and cells assume a new direction of movement.

Zuzana Koledova from Masaryk University in Czech Republic closed this session with a short talk on her current work on the role of fibroblasts on mammary gland morphogenesis. Fibroblasts are known to interact with the mammary gland epithelium through paracrine signaling as well as through extracellular matrix production and remodeling [25]. Zuzana Koledova team currently investigates the potential involvement of mechanical forces exerted by fibroblasts in mammary epithelial branching using mouse models, co-cultures of fibroblast and mammary gland epithelium [26] and time lapse visualization of organoid dynamics and branching.

The student/postdoc session chairs of this year were Elsa Charifou from Institut Pasteur in France and Gunnhildur Asta Traustadottir from University of Iceland in Iceland. They chose the topic of the session, tumor microenvironment, as well as the speakers for their session.

The first talk was given by Jayakumar Vadakekolathu from Nottingham Trent University in UK. His work is focused on the EMT as one of the key steps in the metastatic process. He modelled EMT transition in a cell line model from which clonal progenies with different epithelial and mesenchymal characteristics have been derived [27]. Comparison of expression profiles and proteomics between the different clonal progenies revealed that *NNMT* is the most upregulated gene in the most mesenchymal clone. Data mining and analysis as well as in vitro experiments showed that *NNMT* seemed to be a key regulator of EMT and that *NNMT* expression was associated with tumor grade level in breast cancer. High expression of *NNMT* was correlated with poor relapse free survival, indicating its probable role in promoting metastatic spreading. His findings suggest that inhibition of *NNMT* might be an ideal candidate to target to reduce triple negative breast cancer progression.

The last talk of this session, presented by Ingunn Holen from the University of Sheffield, UK, was focused on the role of the bone microenvironment in regulating breast cancer metastasis. Studies have shown that up to 60% of patients with detectable disseminated tumor cells (DTC) in bone marrow remain relapse-free 6 years later. This implies that tumor cells reaching bone is not in itself sufficient to develop metastasis and that events at the secondary site are key to tumor progression. To study the effect of the bone microenvironment on DTC and metastasis, her team used a mouse model where intracardiac injection of labelled human breast cancer MDA-MB-231 cells formed tumors in the bone and DTCs in bone can then be studied using 2-photon microscopy. She showed that tumor cell homing was not uniform, with the majority of breast cancer cells located in the trabecular areas of the bone. Her work also showed that there may be a limited number of suitable niches available in bone for DTCs to colonise. When hematopoietic stem cells (HSC) were mobilized from their bone niches to the circulation (via injection of AMD3100, an inhibitor of *CXCR4*) prior to the injection of tumor cells, the number of DTC in trabecular regions increased, suggesting that the HSC and the bone metastatic niche overlap [28]. She presented a model of tumor dormancy in bone, allowing comparison of conditions in which DTC will develop in metastasis or remain dormant based simply on the age of mice (which impacts the bone microenvironment) at the onset of the experiment. By expanding the osteoblast number with parathyroid hormone prior to tumor cell injection, she showed that the osteoblasts were important for tumor cell survival and progression but not for DTC homing [29]. She also described studies of the effect of osteoclast activity on DTC by ovariectomizing (OVX) mice. OVX induced rapid bone loss and increased osteoclast activity, which triggered a large increase in growth of DTC to form new colonies in bone, compared to a sham operation. The OVX-induced growth of DTCs was inhibited with anti-resorptive agents, demonstrating tumor growth is driven by osteoclast-mediated mechanisms [30].

In conclusion, the virtual 2021 workshop was a success, with a very wide range of techniques described to obtain highly relevant new observations for breast cancer patient benefit and mammary gland fundamental comprehension. The DeOme prize for the best short talk presentation was awarded to Ana Luisa Correia from Mohamed Bentires-Alj lab in University of Basel, Switzerland. The three poster prizes were awarded to Johanna Englund, from the University of Helsinki, for her study of the role of laminin alpha 5 in the mammary gland, Guillaume Jacquemin, from Silvia Fre’s lab in Institut Curie in Paris, for his work on improving windows for in vivo imaging of the mouse mammary gland, and Hannah Harrison, from University of Manchester, for her work describing a novel explant culture model for studying normal human breast tissue.

The next ENBDC workshop will take place in Weggis, Switzerland on April 28–30, 2022 and will be chaired by Beatrice Howard from the Institute of Cancer Research in London, UK, co-chaired by Jos Jonkers, from the Netherlands Cancer Institute in Amsterdam. The student/postdoc chairs will be Jakub Sumbal, from Masaryk University in Czech Republic, and Hannah Harrison, from University of Manchester.
